# Supplementary material for: Predicting visual function by interpreting a neuronal wiring diagram
Source: Nature. 2024 Oct 2;634(8032):113–23. doi: 10.1038/s41586-024-07953-5 (PMC11446822; doi:10.1038/s41586-024-07953-5)
Supplement: Supplementary file 6 — CRF and ERF predictions for individual TmY4 and TmY9 cells. Analogous to Supplementary Data 3, but for TmY target types. Shown are the top four monosynaptic pathways, the strongest pathway passing through each of the top ten intermediary types (ranking from Extended Data Fig. 7), and the trisynaptic pathway Tm1–TmY–Dm3–TmY (see the section entitled Prediction of spatial normalization). [file 41586_2024_7953_MOESM6_ESM.zip › DataS4/TmY9q/720575940628102824.pdf]

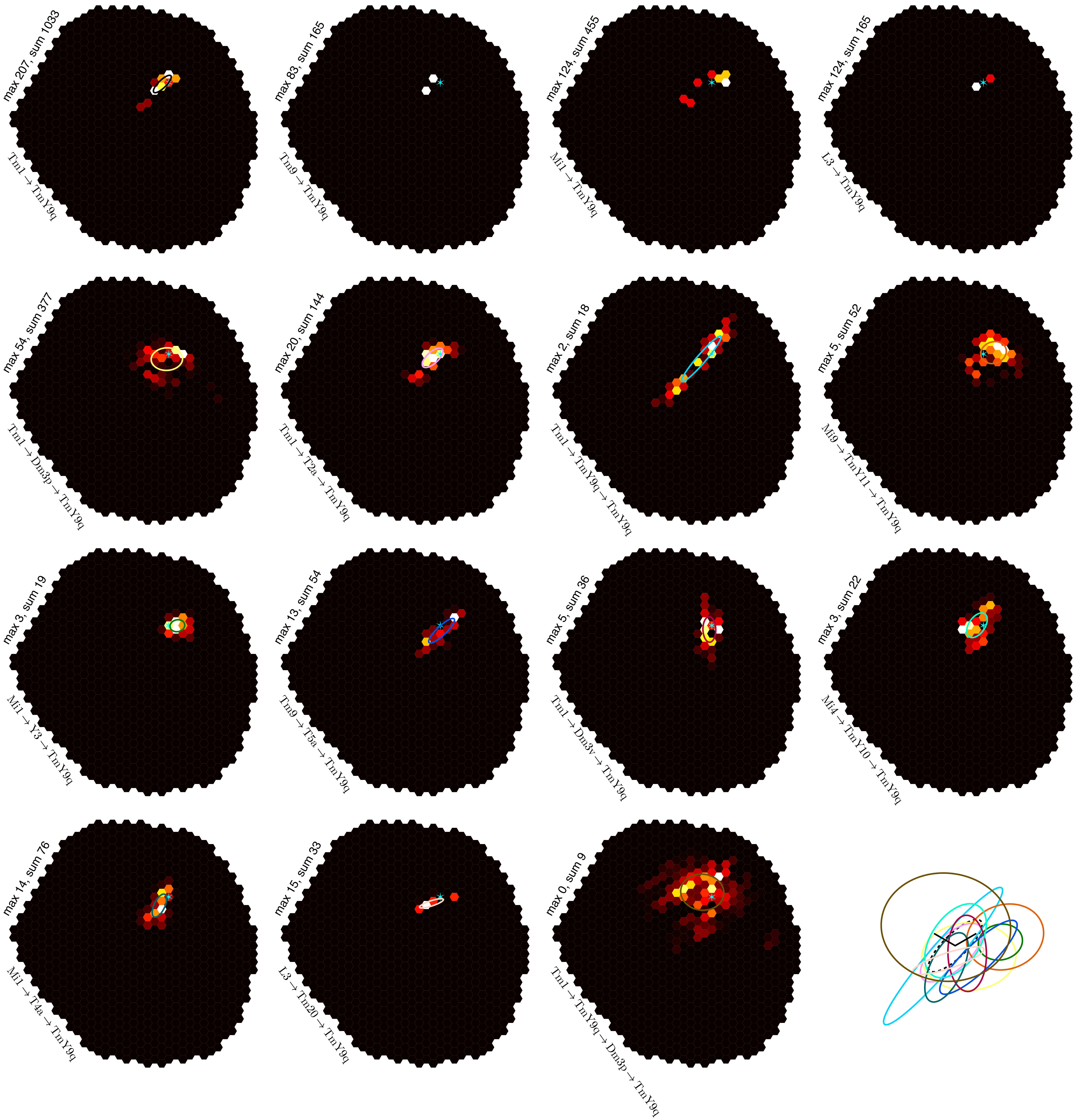

max 207, sum 1033
Tm1 → TmY9q
max 83, sum 165
Tm9 → TmY9q
max 124, sum 455
Mi1 → TmY9q
max 124, sum 165
L3 → TmY9q
max 54, sum 377
Tm1 → Dm3p → TmY9q
max 20, sum 144
Tm1 → T2a → TmY9q
max 2, sum 18
Tm1 → TmY9q → TmY9q
max 5, sum 52
Mi9 → TmY11 → TmY9q
max 3, sum 19
Mi1 → Y3 → TmY9q
max 13, sum 54
Tm9 → T5a → TmY9q
max 5, sum 36
Tm1 → Dm3v → TmY9q
max 3, sum 22
Mi4 → TmY10 → TmY9q
max 14, sum 76
Mi1 → T4a → TmY9q
max 15, sum 33
L3 → Tm20 → TmY9q
max 0, sum 9
Tm1 → TmY9q → Dm3p → TmY9q
